# Dry Liposculpture of the Calves and Ankles—A Novel Technique for Sculpting the Lower Legs

**DOI:** 10.1007/s00266-024-04402-6

**Published:** 2024-09-29

**Authors:** Jeremy Niddam, Noam Castel, Ariel Berl, Avshalom Shalom

**Affiliations:** 1 Department of Plastic Surgery, Meir Medical Center, Kfar Saba, Israel; 2 School of Medicine, Faculty of Medical and Health Sciences, Tel Aviv University, Tel Aviv, Israel

**Keywords:** Dry liposuction, Calves, Ankles, Liposculpture, Tourniquet, Technique

## Abstract

**Background:**

Liposuction of the calves and ankles was historically described as a “taboo” procedure and linked with higher postoperative pain and unpleasing aesthetic results.

**Objectives:**

This study presents a Novel technique for dry liposculpture of the calves and ankles using a tourniquet, as a safe and less painful procedure, which achieves better aesthetic results.

**Methods:**

This single surgeon, retrospective study included 70 women who underwent surgery based on the technique described. Details regarding the surgical outcomes and pain levels were collected, and patients completed 2 years of follow-up.

**Results:**

A mean of 1395 cc fat was aspirated from both legs, combined, and the mean duration of surgery was 98 minutes. No major complications were observed, and postoperative pain levels were low. Pre- and post-surgery pictures were evaluated by 8 certified senior plastic surgeons for aesthetic results.

**Discussion:**

The findings suggest that dry liposculpture is suitable for reshaping the calves and ankles.

**Conclusions:**

This technique is safe, with precise and satisfactory aesthetic results, low levels of postoperative pain, and may take less time to perform than wet liposuction.

**Level of Evidence IV:**

This journal requires that authors assign a level of evidence to each article. For a full description of these Evidence-Based Medicine ratings, please refer to the Table of Contents or the online Instructions to Authors www.springer.com/00266.

**Supplementary Information:**

The online version contains supplementary material available at 10.1007/s00266-024-04402-6.

## Introduction

The leg has become an important aesthetic characteristic of women, and the ankle and calf are considered feminine areas whose appearance is important. This has led women to seek improvement through surgical intervention. Patients are increasingly interested in enhancing their appearance by reshaping their calves and ankles. Some authors consider circumferential liposuction a suitable method for correcting fat deformities in these areas [1–3].

Lipodystrophy of these regions is usually present from early adolescence, and the calf might have a thickened and unshapely appearance. Lilis described these areas as diet and exercise resistant, creating an emotionally frustrating cosmetic deformity, that often made patients look much heavier than they really were [1].

Previous studies on surgical techniques addressing calf and ankle liposuction described the use of various infiltration methods prior to liposuction (in this paper, wet, super wet, and tumescent methods will be generally addressed as a “wet technique”) [3]. Circumferential wet liposuction of the lower leg requires experience to achieve satisfying aesthetic contouring results. The main complications related to this procedure are surface irregularities, persistent edema, postoperative pain, and patient dissatisfaction [4–6].

The aim of the study was to assess the safety and advantages of liposuction of the calves and ankles using the dry liposuction technique with a tourniquet, as performed in our clinic and to present two-year follow-up results.

## Methods

This single surgeon, nonrandomized, retrospective study was conducted from June 2017 to June 2020. The study included female patients who were eligible for surgery using a dry liposuction technique for the calves and ankles while using a tourniquet. All patients were operated on by the senior author in a single surgical center.

Patients with a history of deep vein thrombosis (DVT), pulmonary embolism (PE), diabetes, coronary artery disease or venous insufficiency, or a BMI > 30 kg/m^2^ were excluded from the study.

Patient demographics and surgical parameters, including age, BMI, and smoking status (defined as smoking within 8 weeks before surgery), were documented and stored in a Microsoft Excel spreadsheet (Microsoft Corp., Redmond, WA, USA).

Duration of the procedure was documented according to the tourniquet clock, starting with inflation on the first leg and completed with deflation of the second leg. The total volume of aspirated fat was also documented.

Patient reported pain was documented on postoperative day (POD) 1, 7, and 15, using a visual analogue scale of 0 (no pain) to 10 (worst pain experienced). Major complications were documented and defined as hematoma, infection, and skin necrosis which required surgical intervention, as well as DVT, PE, and paresthesia.

Standardized photographs of patients were obtained before surgery and on POD 90 for comparison and outcome evaluation. Pictures were taken from the frontal view, an angle of 45 degrees between the frontal and lateral views, rear, and 45 degrees between rear and lateral views.

Aesthetic surgical outcomes were evaluated by 8 blinded, certified plastic surgeons, who reviewed the preoperative photographs and those taken 90 days after surgery. They were asked to evaluate the aesthetic results on a scale of 1 to 7, (1—extremely poor, 2—poor, 3—barely acceptable, 4—acceptable, 5—good, 6—very good, and 7—excellent.)

## Surgical Technique

### Preoperative Marking

**Fig. 1:**
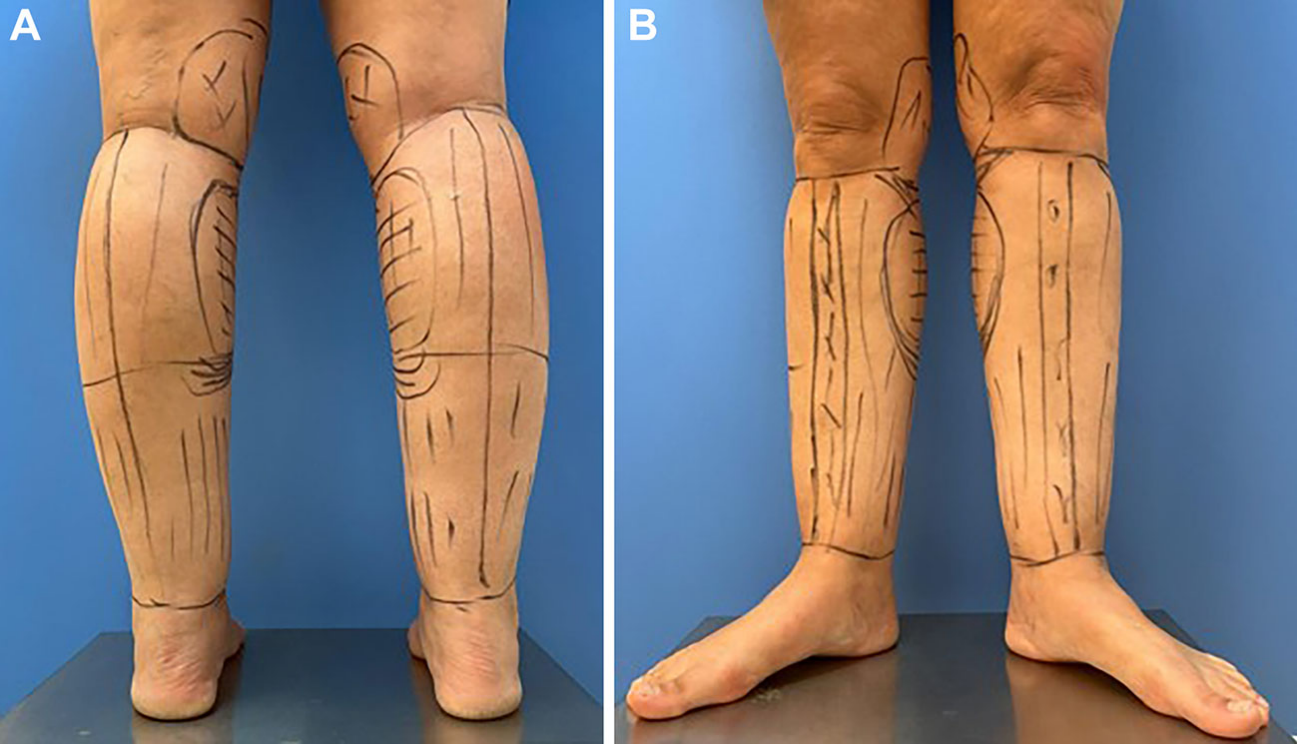
**A** Preoperative marking performed in standing position, areas with deposits of fat are identified. **B** The medial gastrocnemius muscles are marked, and liposuction is usually avoided in this area

Markings were performed with the patient in the standing position. Areas with deposits of fat were identified, and the areas between the Achilles tendon and the medial and lateral malleus were marked for liposuction. The medial gastrocnemius muscles were also identified as shown in Fig. 1.

### Intraoperative Procedure

Surgery is performed under general anesthesia, with the patient in the prone position. The same technique is used on both legs. A TR electronic tourniquet (DCS Medical, LTD, Ra’anana, Israel) is positioned above the knee. The leg is lifted, and veins are drained using an Esmarch bandage. The tourniquet is then inflated to 300 mmHg, and the Esmarch bandage is removed (a short video of the procedure is available).

**Fig. 2:**
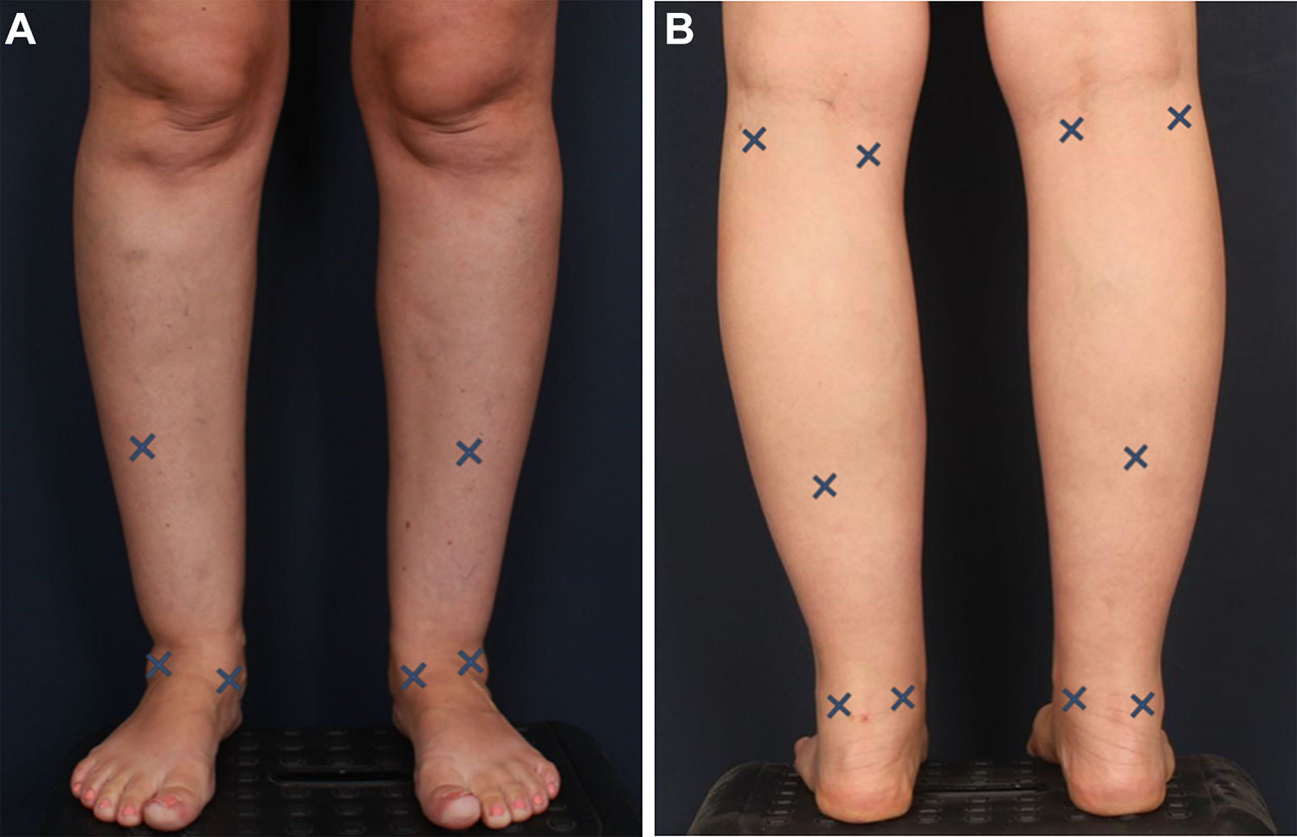
Illustration of the 8 incisions for cannula insertion. **A** 3 anterior incisions, and **B** 5 posterior, as marked

Eight stab incisions are made in each leg, using an 11 blade: four around the ankle, one posterior and one anterior to the mid-calf, and one lateral, and one medial just below the knee (Fig. 2). Power-assisted liposuction is used (Vitruvian ultimate aspirator, Black & Black Surgical, Tucker, GA, USA). Liposuction of the ankle is performed using a 3-mm 3-hole cannula, and the calf is suctioned using a 4-mm 3-hole cannula. While the patient is still in the prone position, liposuction of the anterior calf and ankle is performed while the knee is flexed 90 degrees from the ground, with the foot directed upwards and the toes pointing up. In the area of the ankles, the liposuction is used to its maximum extent, while sparing the Achilles tendon and the lateral and medial malleoli. In the calf area, liposuction of the medial gastrocnemius is minimal, which simulates the appearance of a well-defined muscle and creates an overall athletic shape. After the liposuction is completed, a 4-mm basket cannula is used without suction to equalize the surface. Incisions are sutured using Vicryl rapid 5/0 suture (Ethicon, Somerville, NJ, USA). Patients are discharged on the day of the procedure after anesthesia clearance and several hours of observation.

### Postoperative Care

After completion of the operation, prior to deflation and release of the tourniquet, force 3 level compression socks are fitted above the knee.

Patients are instructed to wear the compression socks for 3 months, 24 hours a day. Prophylactic treatment with subcutaneous enoxaparin (40 mg/day) is administered for 5 days after surgery. Patients are instructed to ambulate as early as possible. Follow-up visits are scheduled on POD 1, 7, and 15 after surgery. Further follow-up visits are scheduled every 3 months for 2 years.

## Results

A total of 70 female patients were included in this study. All patients underwent dry calf and ankle liposuction according to the described technique, between June 2017 and 2020. All 70 patients have completed 2 years of follow-up.

The average age was 34.7 years (range 19–65), with an average BMI of 24.2 kg/m^2^ (range 20-29). Twenty-two patients were active smokers who ceased smoking 4 weeks before surgery. A mean of 1395±645 cc fat (range 500–3000 cc) was aspirated from both limbs combined, and the mean duration of surgery was 98 minutes (range 91-150 minutes). Details of each patient are provided in the Supplementary file.

### Surgical Outcomes

**Fig. 3:**
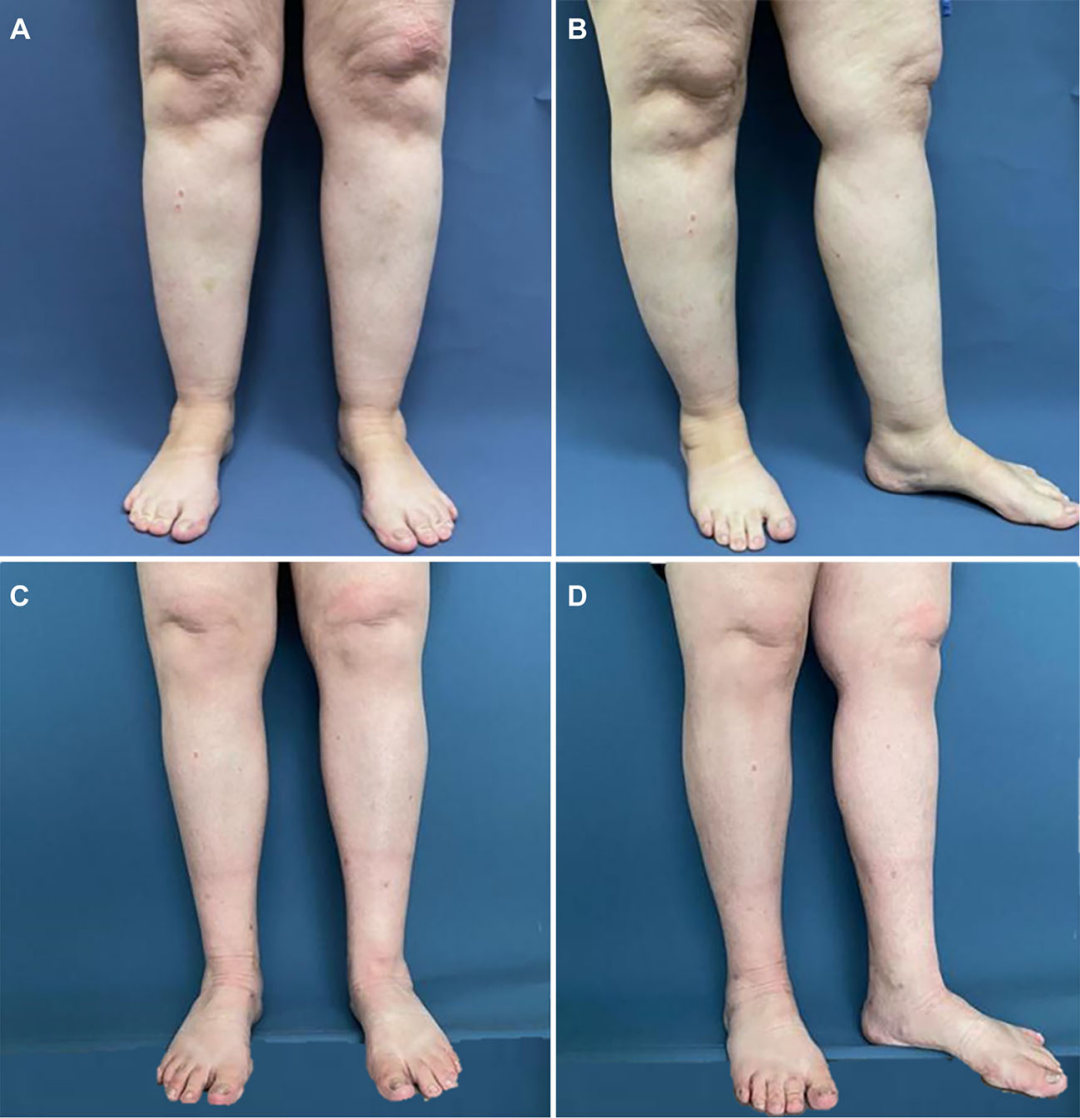
**A**,**B** Preoperative and **C**,**D** postoperative photographs taken 3 months after surgery of a 51-year-old female patient. A total of 2600 cc was aspirated from both legs

**Fig. 4:**
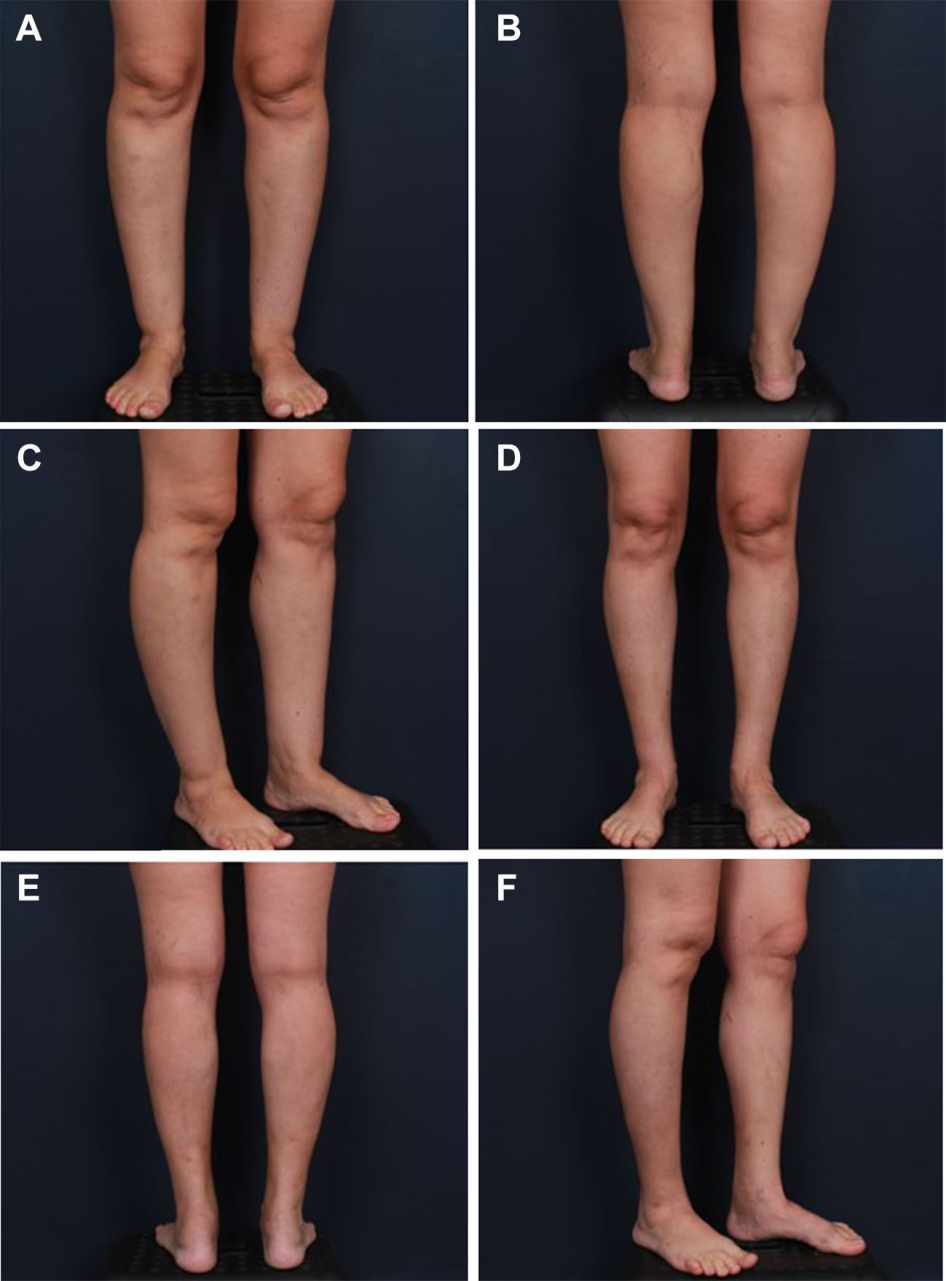
**A**,**B**,**C** Preoperative and **D**,**E**,**F** postoperative photographs taken 3 months after surgery of a 32-year-old female patient. A total of 900 cc was aspirated from both legs

Patients were photographed for documentation using a canon EOS 600D camera. Photographs were taken prior to surgery as shown in Figs. 3A, B and 4A–C and 90 days post-surgery as shown in Figs. 3C, D and 4D–F. The aesthetic results evaluated by 8 plastic surgeons, on a scale of 1 (extremely poor) to 7 (excellent), ranged from 5 to 7, with an average result of 6.2 and a median of 6.

### Follow-Up

The mean postoperative pain level as documented by the 70 patients included in this study was 2.4 on POD 1, 0.9 on POD 7, and 0.4 on POD 15 as shown in Table 1.

**Table 1:** Patient reported postoperative pain in a scale of 0–10

Variable	POD 1	POD 7	POD 15
Range (min–max)	1– 5	0− 3	0− 2
Mean	2.4	0.9	0.4
Median	2	1	0

^*^POD, postoperative day

No major complications, including hematoma, infection, skin necrosis, DVT, or PE, were observed. Among the patients, 21 (30%) had minor ecchymoses which resolved spontaneously a few weeks after the procedure. One patient reported a 10 cm^2^ area of paresthesia over the dorsal area of her right foot on POD 90, which had resolved spontaneously by the next follow-up visit.

## Discussion

The dry liposculpture technique using a tourniquet, as presented in this study, is safe and reproducible, with low post-operative pain scores and very satisfactory aesthetic results.

Historically, liposuction of the calf and ankle has always been considered challenging, causing postoperative pain and requiring long recovery times [4, 5]. The most radical historical documentation regarding liposuction in these areas is from 1920, when Dujarrier attempted to reshape the ankles of a ballerina. This resulted in the inconceivable complication of amputation [7]. It was not until Illouz developed the blunt cannula for liposuction in 1978 that treatment of the calves and ankles was again attempted. Illouz stated that treatment of these regions requires careful attention because of their anatomy of one layer of subcutaneous fat containing dense connective and fibrous tissues and many lymphatic vessels, presenting an operative challenge which may easily lead to irregularities in the surface of the skin [8]. Furthermore, he later described the calf as a taboo area for liposuction because of these anatomical characteristics [9].

Subsequently, Teimourian was probably the first author to briefly mention the potential role of a tourniquet in liposuction, although he infiltrated the tissue prior to fat aspiration [10].

In 1999, Cherif-Zahar [11] described dry liposuction of the legs using a tourniquet and addressed its advantages, such as less post-operative pain and superior aesthetic results. This study described important foundations for the development of liposuction of the calves and ankles. However, for unknown reasons it has not gained enough attention since infiltration prior to liposuction is still a common method for treating the calves and ankles as shown in Table 2

**Table 2:** Patient demographics

Patient	Age	BMI	Comorbidities	Tobacco user	Minor complication
1	34	21	–	0	0
2	23	20	–	0	0
3	46	24	–	0	Ecchymosis
4	33	23	–	1	Ecchymosis
5	24	22	–	1	0
6	27	25	–	0	0
7	21	24	–	0	0
8	54	26	Hypertension	0	Ecchymosis
9	43	25	–	0	0
10	34	22	–	0	0
11	33	23	–	1	Ecchymosis
12	54	25	–	1	0
13	56	26	Hypertension, RA	0	Ecchymosis
14	43	29	–	0	0
15	32	27	–	1	0
16	27	27	–	1	0
17	29	27	–	1	Ecchymosis
18	23	25	–	0	0
19	43	27	–	0	0
20	48	22	–	0	0
21	37	26	–	0	0
22	39	24	–	0	0
23	40	24	–	0	0
24	24	27	–	0	0
25	27	29	–	0	0
26	26	22	–	0	0
27	41	24	–	1	Ecchymosis
28	39	28	–	0	Ecchymosis
29	33	29	–	0	0
30	32	26	–	0	0
31	41	24	–	0	0
32	25	22	–	1	Ecchymosis
33	27	22	–	1	0
34	29	21	–	1	Ecchymosis
35	31	23	–	0	0
36	32	24	–	0	0
37	65	27	Hypertension	0	Ecchymosis Paresthesia*
38	54	26	Hypertension, hyperlipidemia	1	Ecchymosis
39	44	24	–	1	Ecchymosis
40	45	22	–	1	Ecchymosis
41	25	21	–	1	0
42	28	22	–	0	0
43	23	25	–	0	Ecchymosis
44	19	27	–	1	Ecchymosis
45	42	24	–	0	0
46	26	23	–	0	0
47	24	22	–	0	0
48	25	21	–	0	0
49	38	27	–	0	0
50	38	23	–	0	Ecchymosis
51	58	24	–	1	0
52	32	26	–	0	Ecchymosis
53	25	25	–	1	0
54	27	25	–	0	0
55	41	23	–	1	Ecchymosis
56	32	22	–	1	0
57	31	27	–	1	Ecchymosis
58	35	24	–	0	0
59	51	22	Hyperlipidemia	0	0
60	43	21	–	0	0
61	25	25	–	0	0
62	37	28	–	0	Ecchymosis
63	36	26	–	0	0
64	35	24	–	0	0
65	40	23	–	0	Ecchymosis
66	24	22	–	0	0
67	22	24	–	0	0
68	21	21	–	0	0
69	32	22	–	1	0
70	39	21	–	0	0
Average	34.7	24.2	–	–	–
Median	33	24	–	–	–
Range	19–65	20–29	–	–	–

BMI—Body Mass Index (kg/m^2^), RA—rheumatoid arthritis

One patient reported a 10 cm^2^ area of paresthesia over the dorsal area of her right foot on POD 90, which had resolved spontaneously by the next follow-up visit

.

Since Cherif-Zahar’s work, the new concept of high-definition liposculpture (HDL) has emerged. Hoyos et al. described HDL as an artistic approach to body countering, creating not only a slim figure, but also the appearance of highly developed musculature. “An attempt to achieve aesthetically ideal human from revealing underlying anatomical structures…” He characterized HDL as 360° body sculpting which may include fat grafting in addition to liposuction [12, 13].

We believe our study presents a combination of the previously described dry liposuction technique with the newer concept of HDL.

Comparing Cherif-Zahar’s techniques and ours, we focus our preoperative markings and the subsequent liposuction on reshaping and sculpturing the leg, addressing the areas in which suctioning is performed or avoided, and by doing so, we achieve high-definition results. While Cherif-Zahar performed the procedure with the patient in a supine position, we prefer the prone position and bend the knee during the anterior liposuction. This position allows a better approach for shaping the posterior portion of the shin and ankle which demands special attention and in which the surgeon should be able to perform comfortably.

When considering the anatomical characteristics of the lower leg and the increased pain described following liposuction in this area, we believe that infiltration prior to liposuction of the calves and ankles might be the reason why significantly higher pain levels are reported when operating on this region compared to other parts of the body [4, 5].

We believe that infiltration of the calves and ankles causes stretching and pressure in a constricted, cylindrical structure that is surrounded by thick, inflexible skin. Therefore, infiltration prior to liposuction is not recommended because the anatomical features are inherently unique. Since in our technique the surface area is not infiltrated and is highly visible with no blurring effect, the risk of accidental trauma to the underlying muscle from the cannula during suctioning is diminished.

In the present study, the post-operative pain was quite low. Patients reported pain levels ranging from 1 to 4 (on a scale of 0–10) on POD 1 that had already dropped to an average of 0.9 by POD 7, while most patients reported their pain level was 1. On POD 15, the average pain level was 0.4, with most patients reporting no pain.

Regarding the aesthetic results, infiltration of the lower leg prior to liposuction significantly blurs and distorts the surface area. Patients seem to be less satisfied with the results after liposuction of the lower leg and that the main challenge for the surgeon in this procedure is to avoid creating surface deformities [14].

From our experience refining the operative technique and abandoning traditional infiltration practices, we feel that the dry method is preferable when it comes to the surgeon's ability to control the aesthetic result, while avoiding the blurring effect caused by infiltrating the underlying tissue. Indeed, the aesthetic results of our study as graded by blinded plastic surgeons were very satisfactory.

Moreover, while the dry technique can be used in relatively large procedures in which sizable volumes of fat are aspirated (Fig 3), it primarily enables the surgeon to achieve fine and accurate results in patients who need a more minor procedure. Figure 4 shows the pre- and postoperative pictures of a young patient in which the dry procedure enabled reshaping of the calf and ankle, highlighting the area of the gastrocnemius, and sculpturing the lower regions, creating a better contour and shapely appearance. We use 8 stab incisions for cannula insertion on each leg. Although the number of incisions might be considered high, it enables the surgeon to sculpt the lower leg and achieve a high-definition result, which in our opinion could not be achieved with fewer incisions.

Another important advantage of our technique is the minimal to no blood loss due to the use of a tourniquet, which allows aspiration of pure, bloodless fat.

While infusion of a solution containing epinephrine was first used in liposuction to control excessive bleeding by vasoconstriction, we believe that applying this technique universally for all body areas is not warranted.

When addressing the calves and ankles, the use of an Esmarch bandage and a tourniquet is preferable because they allow almost complete control of bleeding. The surgeon can identify the surface area without distortion, and avoiding epinephrine can prevent the unwanted systemic effects of tachycardia and alterations in blood pressure during surgery.

Using the described technique can also save costs, as operating room time is decreased due to avoiding infiltration and the necessary waiting time. However, it should be mentioned that the time to apply the Esmarch bandages, inflate the tourniquet, and place the constrictive dressings at the end of the procedure must be considered. Although we did not compare costs, it is our notion that every surgeon performing liposuction is aware of the time that the infiltration and necessary waiting time might take.

This study had several limitations. Based on our experience, the dry method is highly preferable for calf and ankle liposuction. However, this was a retrospective, single surgeon study and there was no control group for comparison. Another limitation regarding the operative technique was the inability to compare between legs during the procedure due to the compression sock that is applied immediately after completing the operation on one leg before beginning the procedure on the other. This also diminishes the surgeon's ability to achieve the best symmetrical, aesthetic result. Yet, no considerable asymmetries were noted after following the described technique and patient satisfaction was high. Large, multicenter studies are warranted to further assess this technique.

## Conclusions

Based on our experience, we propose dry liposculpture as a suitable method for performing liposuction of the calves and ankles. The technique enables better control, while avoiding surface distortion. Precise aesthetic results were achieved when aspirating both large and small volumes of fat, with minimal blood loss. The dry technique was shown to be safe and reproducible, with highly satisfactory results. It caused low levels of postoperative pain, was less time-consuming, and resulted in superior aesthetic results. The senior author has used and refined this dry liposuction technique for over 6 years and the large cohort, along with reproducible results withstands the test of time. This retrospective study adds to the limited body of literature describing the use of dry liposuction with a tourniquet, for sculpting the lower legs.

## Electronic supplementary material

Below is the link to the electronic supplementary material.

Supplementary file1 (HTM 2 KB)

Supplementary file2 (DOCX 13 KB)

Supplementary file3 (MP4 52256 KB)

## Appendix 1

See Table 2.
