# Correction to: Dry Liposculpture of the Calves and Ankles—A Novel Technique for Sculpting the Lower Legs

**DOI:** 10.1007/s00266-024-04491-3

**Published:** 2024-10-30

**Authors:** Jeremy Niddam, Noam Castel, Ariel Berl, Avshalom Shalom

**Affiliations:** 1 Department of Plastic Surgery, Meir Medical Center, Kfar Saba, Israel; 2 School of Medicine, Faculty of Medical and Health Sciences, Tel Aviv University, Tel Aviv, Israel

**Correction to: Aesthetic Plastic Surgery** 10.1007/s00266-024-04402-6

The original online version of this article was revised to include a supplementary video.

The original article has been corrected.

