# Supplementary material for: Dry Liposculpture of the Calves and Ankles—A Novel Technique for Sculpting the Lower Legs
Source: Aesthetic Plast Surg. 2024 Sep 29;48(23):5074–82. doi: 10.1007/s00266-024-04402-6 (PMC11739234; doi:10.1007/s00266-024-04402-6)
Supplement: Supplementary file 1 — Supplementary file1 (HTM 2 KB) [file 266_2024_4402_MOESM1_ESM.htm]

Error 403 (Forbidden)!!1 

**403.** That’s an error.

We're sorry, but you do not have access to this page. That’s all we know.
