# Supplementary material for: Dry Liposculpture of the Calves and Ankles—A Novel Technique for Sculpting the Lower Legs
Source: Aesthetic Plast Surg. 2024 Sep 29;48(23):5074–82. doi: 10.1007/s00266-024-04402-6 (PMC11739234; doi:10.1007/s00266-024-04402-6)
Supplement: Supplementary file 2 — Supplementary file2 (DOCX 13 KB) [file 266_2024_4402_MOESM2_ESM.docx]

**Video legend**

Dry liposculpture of the lower legs, performed by the first author.

The operation is performed under general anesthesia while the patient is in the prone position.

Esmarch bandages are applied before inflating the tourniquet.

Dry liposuction is performed with no prior infiltration, suctioning over the gastrocnemius muscle is minimal, simulating the appearance of a well-defined muscle.

The knee is flexed, toes pointing up, for suctioning the anterior aspect of the calf.

Suctioning around the ankle is performed while sparing the Achillis tendon area, to achieve a more athletic shape.

As you can see, the aspirated fat in the suction tube is almost completely bloodless
